# Supplementary material for: Chromothripsis during telomere crisis is independent of NHEJ, and consistent with a replicative origin
Source: Genome Res. 2019 May;29(5):737–49. doi: 10.1101/gr.240705.118 (PMC6499312; doi:10.1101/gr.240705.118)
Supplement: Supplemental Material [file supp_gr.240705.118_Supplemental_file_1.zip › contigs/annotated_contigs/DB50/contig.2.DB50_length_298_mean_cov_7.22147651007.docx]

**DB50_length_298_mean_cov_7.22147651007**

```
TACGTCTTTCTCCCAGGCTGGATGCTTCCTGTCCTCAAACATCAGACTCCAGGTTCTTCAGTTTCGAAATTCAGACTGGCTCTCCTTGC
 >chrX:142003967-142004171 + E=2e-90 p=0e+00
TCCTCAGTCTGAAGACAGCCTATTGTGGGACCTTGTGATCATGTGAGTTAATACTTAGTAAACTCCTACATATATATACACATATATAT

GTATGTGTATATATACACATATA|TAT|GTATGTGTATATATACACACATATGTGTGTGTATATATACACACATATGTGTGTGTATATA
                        >chr13:82820438-82820528 - E=6e-23
TATACACACATATGTGTGTGTATATATATACA|C
```
